# Supplementary material for: Environmental mastery mediates relationships between mindsets and well-being
Source: PLoS One. 2025 Jul 2;20(7):e0326997. doi: 10.1371/journal.pone.0326997 (PMC12221179; doi:10.1371/journal.pone.0326997)
Supplement: Attachment — Submitted filename: PONE-D-24-45317-R1-Response to Reviewers.docx [file pone.0326997.s002.docx]

Reviewers' comments:

Reviewer's Responses to Questions

Comments to the Author

1. Is the manuscript technically sound, and do the data support the conclusions?

Reviewer #1: Yes

2. Has the statistical analysis been performed appropriately and rigorously?

Reviewer #1: I Don't Know

3. Have the authors made all data underlying the findings in their manuscript fully available?

Reviewer #1: Yes

4. Is the manuscript presented in an intelligible fashion and written in standard English?

Reviewer #1: Yes

5. Review Comments to the Author

Reviewer #1: Thank you for the opportunity to review this manuscript. While the topic is of interest and importance, I regret to say that I cannot support the publication of the paper in its current form. Below, I provide detailed feedback outlining significant concerns that must be addressed for the manuscript to be considered for publication elsewhere.

The theoretical foundation of the manuscript is weak. It is unclear whether the authors conducted a thorough literature review. For example, the relationship between mindset and achievement is well-documented as being mediated by other factors, such as motivation, grit, and self-efficacy (e.g., Zhao et al., 2024). However, the manuscript implies that these mediational relationships have rarely been studied, which is inaccurate. A more comprehensive review of the literature and clearer articulation of the study's unique contribution are necessary.

Reply: We thank the reviewer for bringing our attention to the Zhao et al. 2024 article. It was published just before we submitted this paper. We read the Zhao article and found no citations to studies that found mediational relationships involving mindsets and some outcome. One study, Hassan et al. examined such relationships and found none. We also found another article, Zhao et al. 2023 (different group of authors) that examined core self-evaluation as a mediator. We cited both of these papers in the revision (introduction and discussion), and we added a recently published paper from our lab about the mediation of relationships between mindsets and well-being. We should note that Zhao et al. 2024 did not always make clear if the studies they cited examined mediation per se or if the results of the studies were consistent with the mediational relationships they discussed. Upon closer examination, it was invariably the latter.

Regardless, we did find some other studies that examined mediational relationships that involved mindsets. For various reasons, none of these studies were directly relevant to ours, and so they were not included in the introduction. Nevertheless, they are relevant to the general topic, and so we have added a section to the discussion in which we discuss these other approaches.

Several key terms, such as "treatment value" and "entity orientation" (p. 4), are introduced without sufficient explanation or context. Providing definitions and background for these terms is crucial for clarity.

Reply: The terms we used were taken directly from the two articles that were cited, and in our opinion, represent commonly used terms. Nevertheless, we have edited this section of the text.

Additionally, the inclusion of correlation coefficients from Burnette et al. on the same page seems unnecessary unless their relevance to the current study is explicitly explained.

Reply: We included the correlations because they were from a meta-analysis, and therefore, we thought they provided a useful context for understanding the strength of the relationships between mindsets and outcomes relevant to mental health and well-being.

Furthermore, the phrase "a series of studies" lacks specificity. If the authors are referring to multiple studies, these should be listed or described. Simply citing a single study is inadequate to substantiate the claim.

Reply: The citation is to a paper that presents the results of four studies. This is why the references follows the phrase “In a series of studies.” We did not cite Howell at al. as an example of such a study in which case the reviewer’s objection would be well-founded; rather, we cited Howell et al. as the source for this.

The manuscript lacks critical details about the study methods, which significantly undermines its credibility.

Reply: In the absence of what constitutes “critical details,” we are somewhat at a loss as to how to reply to this critique. Nevertheless, we tried to intuit what the reviewer found lacking, and we have edited the revision based on our intuition, albeit in a limited fashion.

There is no dedicated data analysis section, leaving readers unable to evaluate how the results were derived. The authors should provide structured and transparent information about their study design, data collection, and analysis plan.

Reply: We have included a section “Overview of analyses” at the beginning of the results section.

We should note that we provided descriptions of the participants, how the data were collected, the measures we used, the scales, and the analyses we conducted. In the absence of any specific suggestions (aside from a request for an overview of the analyses), we are at a loss as to how to respond to this comment.

Moreover, we will assume that by mentioning that we need to be more transparent that the reviewer is not suggesting that we violated any ethical standards by not disclosing pertinent details but is simply requesting more detail (of some kind). For better or worse, in the present day “transparency” has taken on a variety of meanings and connotations.

Additionally, the psychometric properties of the measures used in the study are not addressed. Can the validity and reliability of these measures be assumed? If not, how was this assessed? Without such information, the results are difficult to evaluate.

Reply: It is difficult to know how to address the reviewer’s concerns about the psychometric qualities of the measures. The reliabilities for all measures were presented in Table 1 in the initial submission. Moreover, these reliabilities were described in the text on p. 12: “We estimated reliability using McDonald’s omega. According to the guidelines suggested by Shrout [32], all measures had at least moderate reliability (.72 to .80), and most had substantial reliability (.81 and above).” We are at a loss as to how to make this clearer.

In terms of validity, all the measures we used are in widespread use and have been validated in published studies. We used measures with demonstrated psychometric properties. An examination of our references will show that the scales we used were described in articles published in reputable journals, and these articles included validity analyses. Accordingly, we do not think that we need to revisit the issue of validity in our paper. This is why validation studies are conducted and published.

Another major concern is the lack of attention to potential Type I error inflation due to multiple tests. Have any corrections (e.g., Bonferroni adjustments) been applied? If not, this is a significant limitation that compromises the integrity of the findings.

Reply: Good point. As described in the revision, to address this issue we conducted follow-up Sobel tests of the mediation effects. Sobel tests produce z-ratios that can be evaluated in terms of p-values. As we note in the revision, the p-values for the mediation effects were all significant at p < .000015 or beyond, which we believe puts to rest concerns about inflated alpha levels.

In its current form, the manuscript lacks the rigor and clarity required for publication. Addressing the above issues would require substantial revisions, including a more thorough literature review, clearer definitions and context, and detailed reporting of study methods and analyses. I appreciate the authors' efforts but must recommend rejection.

Thank you for considering my feedback.
