# Supplementary material for: Environmental mastery mediates relationships between mindsets and well-being
Source: PLoS One. 2025 Jul 2;20(7):e0326997. doi: 10.1371/journal.pone.0326997 (PMC12221179; doi:10.1371/journal.pone.0326997)
Supplement: Attachment — Submitted filename: PONE-D-24-45317-R2-Response to Reviewers.docx [file pone.0326997.s003.docx]

**Reviewer #1:** Thank you for your thoughtful and detailed revision. I appreciate the work you’ve done to improve the manuscript, and I can see that it's moving in a good direction. Your responses to the initial feedback were helpful, and the revised version reflects meaningful progress.

At this point, I recommend a major revision to further strengthen the clarity and rigor of the analysis and interpretation.

One area that still needs attention is the construct validity of the outcome measures. My earlier comment on psychometric properties may not have been clear, and I apologize for that. In addition to reporting reliability (e.g., internal consistency), it’s important to consider whether the measures are valid representations of the constructs they intend to capture. If item-level data are available, I encourage you to conduct and report basic checks—such as confirmatory factor analyses—to support the use of the measures. If that’s not possible due to data limitations, it would be helpful to state this and acknowledge it as a limitation.

Reply: It is difficult to know how to respond to this comment. The reviewer mentioned “If item‑level data are available…” We presented reliabilities, which require item level data. Moreover a quick click to the OSF site would reveal a data file with item level data (as described in the paper).

More important, we are not certain what the reviewer means by suggesting that confirmatory factor analysis can be used to establish validity. For example, the ten items of the Rosenberg Self-esteem scale (RSE) are manifest variables of a single latent factor, something that has been confirmed countless times and is also reflected in its high reliability, .88 in the present study. Nevertheless, the results of such a CFA cannot be used to claim that the RSE is a measure of self-esteem, verbal intelligence, or any other construct. Demonstrating validity requires examining relationships between measures of a target construct and measures of other constructs that are assumed to be related to the target construct. Admittedly, examining such relationships can be done using CFA (or simple correlations), but this requires measures of **other** constructs to which the target construct is assumed to be related.

We specifically chose constructs that had measures with demonstrated validity. This can be readily verified by examining the citations we included (and scores of others). We (and numerous other scholars) have used these measures in countless studies. For example, the Rosenberg Self-Esteem scale has been described as the most frequently used measure in personality and social psychology, and its validity is assumed. If the validity of a measure would need to be demonstrated every time a measure was used this would slow progress and also render useless journals such as Psychological Assessment.

Our study was not designed to examine/establish the validity of the measures we used. As we mentioned previously, we chose measures with known psychometric properties, i.e., reliability and validity. There is no way for us to confirm the validity of these measures, nor do we think that not being able to confirm their validity is a limitation. Nevertheless, the reviewer’s concerns may represent the concerns of some readers, and so we have included a brief introduction to the Measures subsection that addresses this issue.

Also, I noticed the mention of the Sobel test in relation to controlling Type I error. Just to clarify, the Sobel test is not intended for that purpose, and its assumptions may not hold in many mediation contexts. Your use of bootstrapping is a more appropriate approach and aligns well with current best practices. However, since you're conducting multiple mediation models, I suggest considering a more conservative approach to confidence intervals—such as using a 99% CI instead of 95%, following a Bonferroni-type adjustment. I marked my recommendation as a major revision since this change in confidence level may impact the significance of your findings.

Reply: The Sobel test provides a traditional (NHST) p-value of the indirect effect, which can be corrected using various methods, something that is difficult with inferences based on CIs. We did not intend the Sobel tests to control for Type I error per se; rather, we thought that by demonstrating that the p-values for the Sobel test were highly significant that this would eliminate concerns about multiple tests. Apparently, it did not. Given this, as per the reviewer’s suggestion, we changed the CI to 99%, and the 99% CIs are presented in Table 2 in the latest revision. We deleted the paragraph describing the Sobel test.
